# Identification and verification of m6A-related miRNAs correlated with prognosis and immune microenvironment in colorectal cancer

**DOI:** 10.1097/MD.0000000000035984

**Published:** 2023-11-17

**Authors:** Xinze Qiu, Da Chen, Shanpei Huang, Ni Chen, Jiangni Wu, Shengmei Liang, Peng Peng, Mengbin Qin, Jiean Huang, Shiquan Liu

**Affiliations:** a Department of Gastroenterology, the Second Affiliated Hospital of Guangxi Medical University, Nanning, China.

**Keywords:** colorectal cancer, microRNAs, N6-methyladenosine, prognostic model, tumor immune microenvironment, tumor mutational burden

## Abstract

It’s well known that N6-methyladenosine (m6A) modification is the most abundant modification in multiple RNA species. miRNAs play important roles in m6A modification and are closely related with occurrence and development of colorectal cancer (CRC). Thus, the aim of this study was to identify the prognostic value of m6A-related miRNAs and explore the correlation between the miRNAs and immune microenvironment in CRC. The differentially expressed m6A regulators and differentially expressed miRNAs between CRC tissues and adjacent normal tissues were identified based on TCGA dataset, and the m6A-related miRNAs were screened. The CRC patients from TCGA were randomized (1:1) into training set and validation set, and the risk score was established in the training set. Next, risk score was verified in the validation set and GSE92928 from GEO datasets. Besides, the relationship among tumor mutational burden, immune microenvironment and risk score were analyzed. What’s more, RT-qPCR were used to explore the expression levels of the miRNAs in risk score between SW480 and SW620. A total of 29 m6A-related miRNAs were screened out, and a 5-differentially expressed miRNAs risk score was established. Kaplan–Meier analysis and ROC curves revealed the risk score could predict the prognosis of CRC, accurately. Similarly, the patients in the high-risk group had shorter overall survival in GSE92928. The risk score was relevant with the tumor mutational burden and immune infiltration, and the expression of HAVCR2 was significant difference between 2 risk groups. The expression levels of miR-328-3p, miR-3934-5p, miR-664b-5p and miR-3677-3p were down-regulated in SW620 compared with SW480, only the expression level of miR-200c-5p was up-regulated in SW620. The findings provided the new insights into the correlation between miRNAs and m6A regulators. The m6A-related miRNAs could predict the prognosis of CRC and provide the valuable information of immunotherapy in CRC patients.

## 1. Introduction

As one of the most common malignant cancer, colorectal cancer (CRC) is the third most frequently diagnosed cancer and the second cause of tumor related deaths worldwide according to the global cancer statistics.^[1]^ Adenocarcinoma is the most prevalent subtype and accounts for more than 95% in CRC. In recent years, the treatment of CRC has made great progress, including surgical resection, systemic therapy, targeted therapy, immunotherapy and so on. Despite these advances, it is disappointing that the prognosis of advanced CRC remains poor.^[2]^ The AJCC TNM staging system is usually used to evaluate the prognosis of CRC, but it can’t be ignored that the staging system couldn’t provide proper prognostic prediction in some cases.^[3]^ Therefore, it is essential to identify novel biomarkers to stratify the prognostic risk and find potential therapeutic targets of CRC.

N6-methyladenosine (m6A) modification was first detected in 1970s, which is the methylation of the sixth N atom of adenosine in mRNAs or ncRNAs.^[4]^ As the most abundant RNA modification in eukaryotes, m6A modification has gradually become a research hot point. The abundance of m6A modification is regulated by m6A readers, writers, and erasers, which could mediate RNA splicing, translation, stability and so on.^[5]^ Up to now, m6A modification has been found to play important roles in cancer pathogenesis and drug resistance.^[6]^ METTL3 has been reported to be an oncogene in CRC cells, which could activate the m6A-GLUT1-mTORC1 axis and facilitate tumor progression.^[7]^ Additionally, m6A reader IGF2BP3 could regulate colon cancer cell cycle and angiogenesis via the m6A modification of CCND1 and VEGF.^[8]^

MicroRNAs (miRNAs) belong to the noncoding RNA family, which contain 19-25 nucleotides.^[9]^ Most of miRNAs could combine with target mRNAs and lead to degradation or translational repression of target mRNAs.^[10]^ Studies have uncovered miRNAs took part in almost all biological process of cancer, such as apoptosis, proliferation, metastasis and immune microenvironment.^[11–13]^ Additionally, increasing evidences showed miRNAs could be regard as prognostic biomarkers in cancers. Remarkably, m6A modification has been reported to be essential in ncRNA splicing and maturation, and involved in carcinogenesis and tumor progression.^[5]^ The study reported that METTL3 could methylate pri-miR-1246 and facilitate the maturation of pri-miR-1246 resulting in CRC metastasis.^[14]^ However, there is still little research to explore the potential mechanism among m6A modification and miRNAs in CRC. Besides, the prediction value of m6A-related miRNAs remains to be elucidated.

In present study, the m6A-related miRNAs were identified and a novel risk score was established based on the Cancer Genome Atlas (TCGA). Tumor mutational burden (TMB) and immune checkpoint genes were found to be associated with risk score. The relationship between the risk score and immune microenvironment was also assessed with ESTIMATE and CIBERSORT algorithm. Otherwise, drug resistance between different risk score groups were further explored.

## 2. Materials and Methods

### 2.1. Data collection

The date of miRNA sequencing, mRNA sequencing (FPKM value), mutation expression, and corresponding clinical information of CRC were acquired from TCGA. The mRNA sequencing was transformed into log2(TPM+1). A total of 25 m6A modification regulators were selected from previous research including 8 writers (METTL3, METTL14, METTL16, WTAP, VIRMA, ZC3H13, RBM15, and RBM15B), 2 erasers (ALKBH5 and FTO), and 15 readers (YTHDC1, YTHDC2, YTHDF1, YTHDF2, YTHDF3, HNRNPC, FMR1, LRPPRC, HNRNPA2B1, IGFBP1, IGFBP2, IGFBP3, RBMX, LAVL1, and EIF3B).

### 2.2. Identification of m6A-related miRNAs

**Figure 1.**
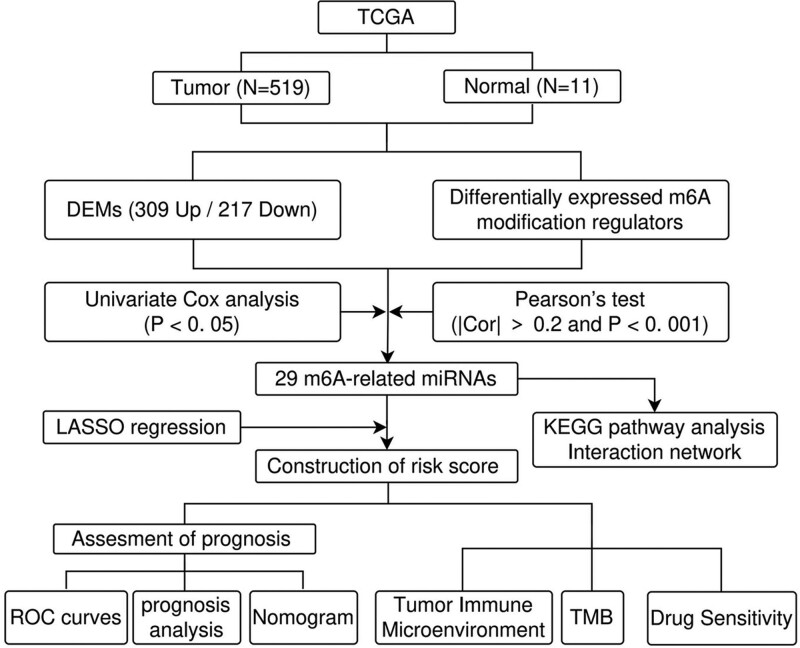
Workflow of the whole study. DEMs = differentially expressed miRNAs, TMB = tumor mutation burden.

The workflow was showed in Figure 1. To identify the differentially expressed miRNAs (DEMs) between CRC and normal colorectal tissues, the “edgeR” package was utilized with the following screening criteria: |log2 fold change| >1 and *P* value < .05 (FDR adjustment); the average expression level no less than 1. Besides, the expression levels of m6A modification regulators were also compared between CRC and normal colorectal tissues. The correlation between DEMs and the differentially expressed m6A modification regulators was evaluated via Pearson’s test, and the m6A-related miRNAs were identified with the screening criteria of Cor > 0.2 and *P* < .001.

### 2.3. Construction of risk score

In order to select the survival-related miRNAs, univariate Cox regression analysis was applied on m6A-related miRNAs, and the acquired miRNAs were adopted into the next analysis. To explore the mechanisms of the miRNAs in CRC, Kyoto Encyclopedia of Genes and Genomes (KEGG) enrichment analysis was used via the website tool DIANA-mirPath v.3 (http://dianalab.e-ce.uth.gr/html/mirpathv3/).

The expression profiles of m6A-related miRNAs and corresponding clinical information were divided into training set and validation set randomly with a 1:1 ratio. The miRNA expression profiles were normalized by log2 before analysis. The least absolute shrinkage and selection operator (LASSO) cox regression analysis was performed to construct of risk score in training set via “glmnet” package.^[15]^ The risk score was calculated by follow formula: Risk score = exprDEM_1_*coefficientDEM_1_+ exprDEM_2_*coefficientDEM_2_ +…+ exprDEM_n_*coefficientDEM_n_. After that, all CRC patients could be stratified into high-risk group and low-risk group based on the median risk score of the training set. What’s more, ROC curves were applied to evaluate the predictive value via “timeROC” package, and Kaplan–Meier survival curves were applied to compare survival difference between high-risk group and low-risk group via “survival” package. Besides, GSE92928 cohort was adopted as a validation set, which contained 165 CRC patients from GEO datasets.

To estimate the independent prognostic value of clinical data and risk score of CRC, univariate and multivariate Cox regression analyses was used in all CRC patients. Besides, hazard ratio (HR) and 95% confidence interval (CI) were also calculated. Furthermore, to improve the prediction power, the nomogram was constructed via “rms” package, including age, gender, TNM stage and risk score.^[16]^ ROC curves and calibration curves were performed to assess the discrimination and deviation of nomogram, respectively.

### 2.4. TMB and tumor immune microenvironment

TMB is defined as the total number of mutations per million bases and was reported to be related with immune checkpoint inhibitors.^[17]^ The patients with higher TMB may indicate better effects of immunotherapy for tumor. The relationship between TMB and risk score was analyzed via Spearman correlation test, and the level of TMB were compared between high-risk group and low-risk group. The expression of the immune checkpoint genes between differential risk groups were also compared using the Wilcoxon’s test.

To explore the correlation between degree of immune cell infiltration and risk score, ESTIMATE algorithm was performed to calculate Immune score, Stromal score and Estimate score using “estimate” package. Additionally, the proportion of 22 marked immune cell subtypes in CRC patients was also calculated via “CIBERSORT” package. Next, the Wilcoxon’s test was conducted to compared the differences of 22 immune cell subtypes between high-risk group and low-risk group. And Spearman correlation test was used to evaluate the relationship between risk score and immune cell subtypes.

### 2.5. Drug sensitivity of risk score

Considering the potential of risk score to predict drug sensitivity, the half-maximal inhibitory concentration (IC50) of the selected drugs was compared between high-risk group and low-risk group via “pRRophetic” package.^[18]^

### 2.6. Total RNA extraction and quantitative real-time PCR

To explore the roles of the miRNAs in CRC metastasis, the expression of the miRNAs was compared between SW480 and SW620 cells, which were isolated from primary tumor and metastatic tumor in abdomen of single CRC patient, respectively. Total RNA was isolated from the cell lines using Total RNA Extractor (No. B511311; Sangon Biotech, Shanghai, China). Reverse transcription reactions were performed using miRNA First Strand cDNA Synthesis Tailing Reaction Kit (No. B532451; Sangon Biotech). The quantitative real-time PCR experiment was performed using a MicroRNAs qPCR Kit (SYBR Green Method) (no. B532461; Sangon Biotech) on an CFX96 Real-Time PCR Detection System (Bio-Rad).^[19]^ The expression of the miRNAs was normalized with U6. The specific primers for the miRNAs were from Sangon Biotech. The result was analyzed using the relative 2^−ΔΔC*t*^ method.

### 2.7. Statistical analysis

All statistical analysis was conducted by R language 4.0.3 (https://www.r-project.org/). Student *t* test and the Wilcoxon’s test was used in continuous variables as appropriate. Kaplan–Meier survival curves were analyzed using log-rank test with “survival” package. The cutoff criteria of statistical significance was set as *P* value < .05.

## 3. Results

### 3.1. Identification of m6A-related miRNAs

**Figure 2.**
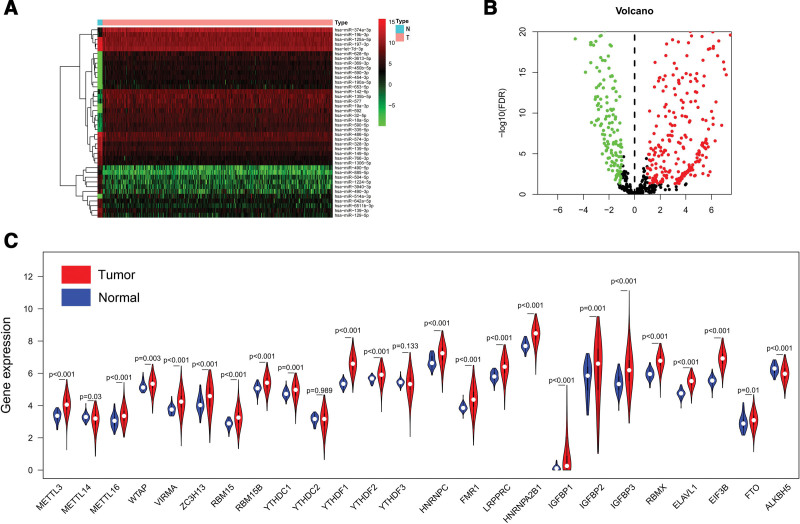
(A and B) Heatmap and Volcano plot showed the differentially expressed miRNAs between tumor and adjacent normal tissues. (C) The expression of 25 m6A modification regulators between tumor and adjacent normal tissues.

After differential expression analysis, a total of 526 DEMs were selected (309 upregulated and 217 downregulated) between CRC and normal colorectal tissues in the TCGA dataset (Fig. 2A and B). As the same time, 23 m6A modification regulators were found to be significantly different between CRC and normal colorectal tissues (21 upregulated and 2 downregulated) (Fig. 2C). To investigate the relationship between DEMs and m6A modification regulators, Pearson’s test was used, and 311 m6A-related miRNAs (Cor > 0.2 and *P* < .001) were screened out finally.

### 3.2. Development and validation of risk score

**Figure 3.**
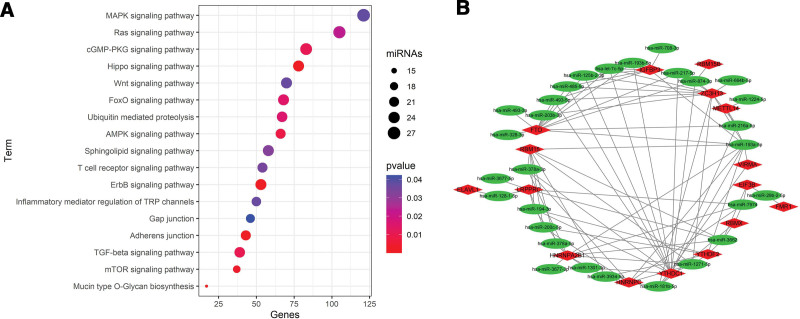
(A) KEGG enrichment analysis was used based on website tool DIANA-mirPath v.3. (B) Interaction network showed the correlation among m6A modification regulators and m6A-related miRNAs. The red diamonds meant m6A modification regulators and the green ellipses meant miRNAs.

A total of 29 m6A-related miRNAs were found to be related with survival via univariate Cox regression analysis. These miRNAs may play important roles in m6A modification of CRC, so KEGG pathway analysis was used and the results showed that these miRNAs were enriched in MAPK signaling pathway, Ras signaling pathway, Wnt signaling pathway, Hippo signaling pathway, TGF-β signaling pathway and so on (Fig. 3A). Besides, network relationship between DEMs and m6A modification regulators was visualized by Cytoscape software (version:3.8.0) (Fig. 3B).

**Figure 4.**
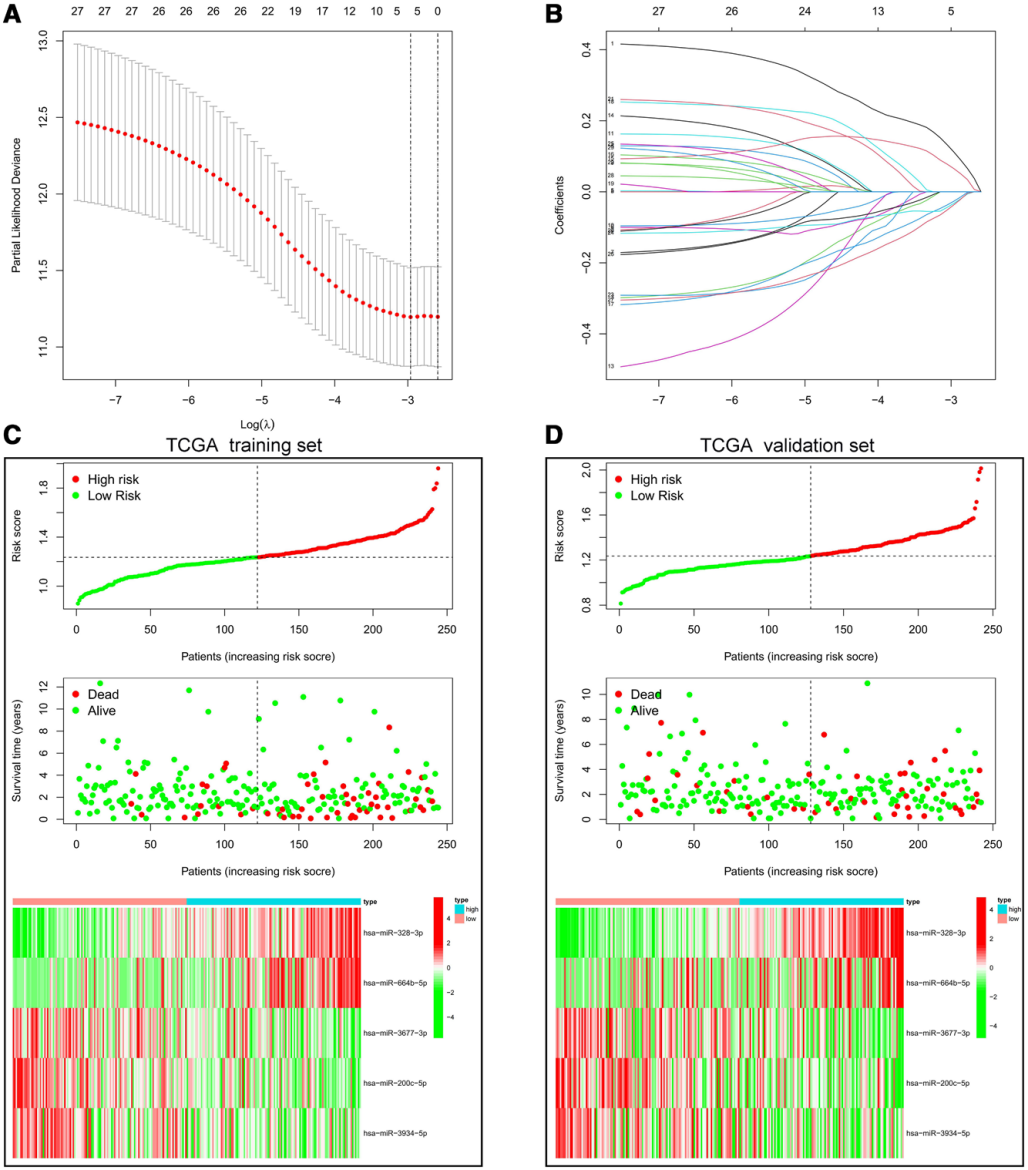
(A) The optimum lambda value was selected in LASSO cox regression analysis. (B) Change of coefficients with different lambda value in the model. (C and D) Risk score, survival time and heatmap of 5 miRNAs between high-risk group and low-risk group in training set and validation set.

**Figure 5.**
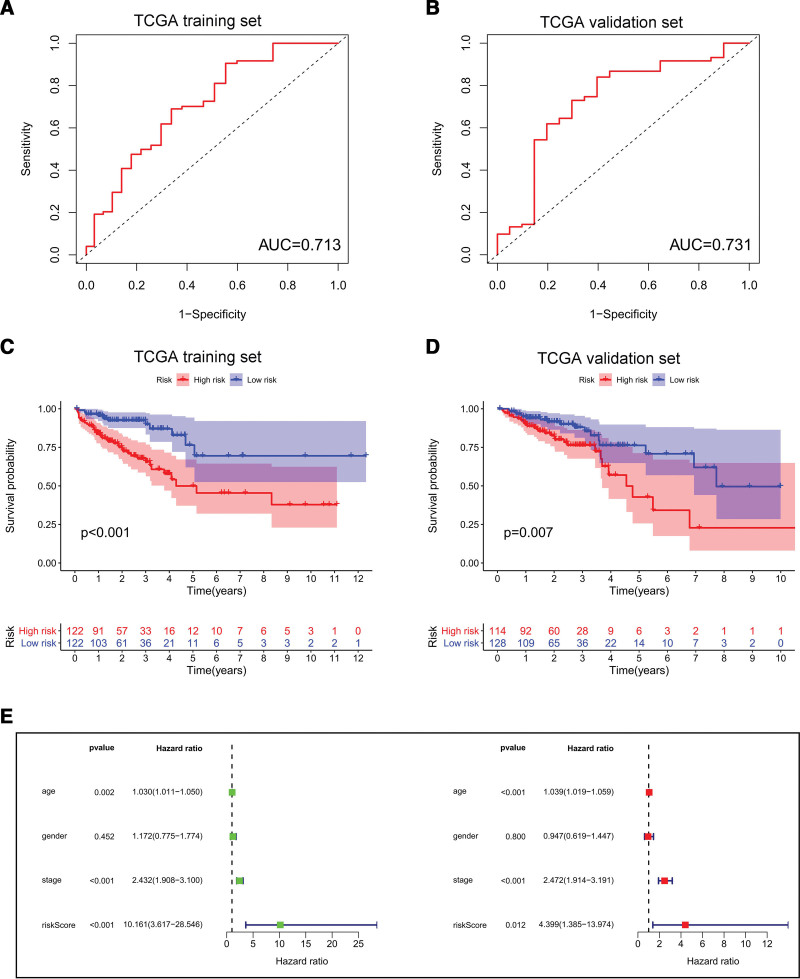
(A and B) ROC curves of risk score based on the 5 miRNAs to predict 5-yr survival rate in training set and validation set, respectively. (C and D) K–M survival curves of different risk groups were performed in training set and validation set, respectively. (E) Univariate and multivariate Cox regression analyses were used in all CRC patients. CRC = colorectal cancer, K–M, Kaplan–Meier.

We screened these miRNAs by the LASSO Cox regression and only 5 miRNAs were selected into the model in training set (Fig. 4A and B). Subsequently, risk score was constructed as follows: Risk score = (0.1120*hsa-miR-328-3p) + (0.0653*hsa-miR-664b-5p) + (−0.0266*hsa-miR-3677-3p) + (−0.0325*hsa-miR-200c-5p) + (−0.0368*hsa-miR-3934-5p). Based on the median of risk score in training set, all CRC patients were divided into high-risk group and low-risk group. In order to exhibit the relationship between risk score and survival status, risk distribution and expression pattern of 5 miRNAs were performed in training set and validation set (Fig. 4C and D). Additionally, time-dependent ROC curves were used to show the accuracy of risk score in survival prediction. The 5-year AUC of training set and validation set were 0.713 and 0.731, respectively (Fig. 5A and B), which implied that risk score exhibited good performance in predicting survival. What’s more, high-risk group had a shorter survival time than low-risk group in both sets (all *P* < .05) (Fig. 5C and D). Similarly, in the external validation, the survival time of patients in the high-risk group were shorter than that of patients in the low-risk group in GSE92928 cohort (Figure S1, Supplemental Digital Content, http://links.lww.com/MD/K663).

To investigate the independent risk factor of all CRC patients, univariate and multivariate Cox regression analysis were used. As is shown in Figure 5E, univariate Cox regression analysis indicated that risk score was significantly associated with overall survival (HR = 10.161, 95% CI = 3.617–28.546, *P* < .001). Age and clinical stage were also related to the overall survival. Multivariate Cox regression analysis showed that risk score still remained independent (HR = 4.399, 95% CI = 1.385–13.974, *P* = .012), which indicated that risk score based on the 5 miRNAs was an independent adverse prognostic factor in CRC.

### 3.3. Establishment of nomogram

**Figure 6.**
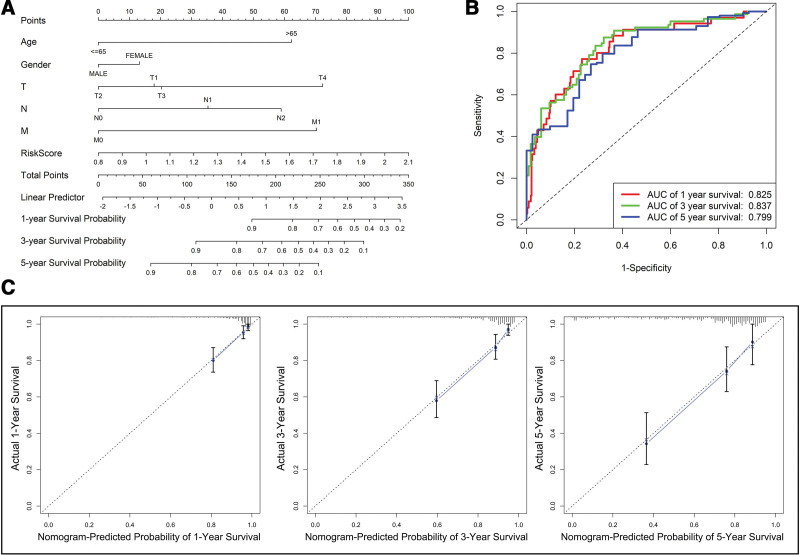
(A) Construction of nomogram for predicting the prognosis of CRC patients. (B) ROC curves of risk score to predict the 1-, 3-, and 5-yr survival times. (C) Calibration curves showed the deviation of nomogram in 1-, 3- and 5-yr survival probability, respectively. CRC = colorectal cancer.

To make risk score more applicable in the clinic, nomogram was established using risk score and clinicopathological parameters, which could provide a better prognostic evaluation for the individual patient. The nomogram contained age, gender, TNM stage and risk score, and risk score was the largest contributors to prognosis compared with the others (Fig. 6A). The AUC of risk score to predict 1-, 3-, and 5-year overall survival was 0.825, 0.837 and 0.799, respectively (Fig. 6B). Besides, Calibration curves were used to evaluate the deviation between prediction and actual observation in 1-, 3-, and 5-year overall survival. As the calibration curves showed, the nomogram could estimate prognosis of CRC patients, accurately (Fig. 6C).

### 3.4. TMB analysis of the risk score

**Figure 7.**
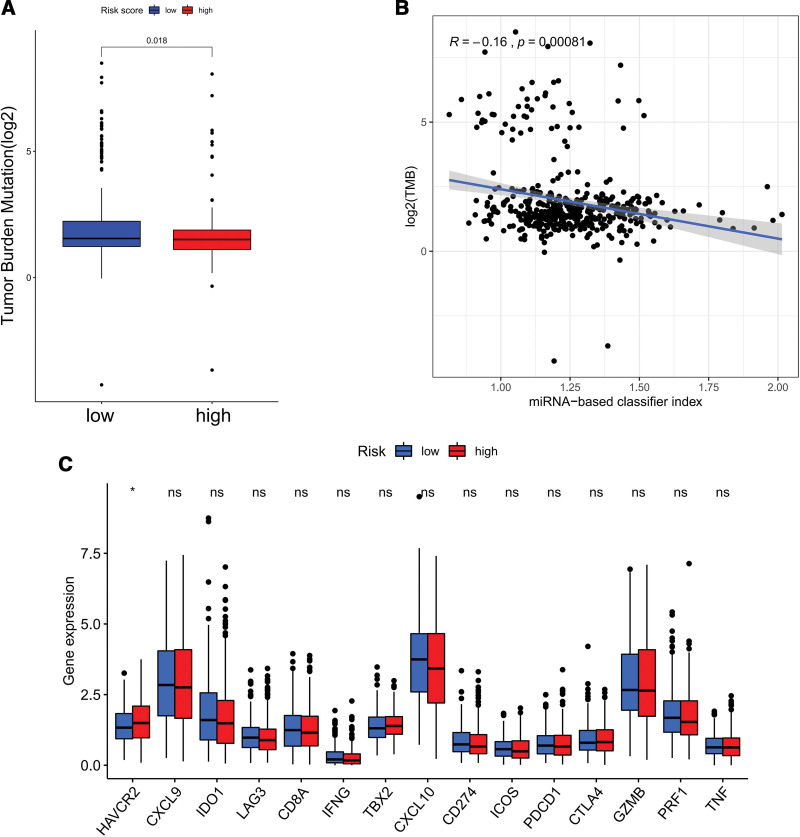
(A) Comparison of TMB levels between high-risk group and low-risk group. (B) The correlation among TMB levels and risk score. (C) The differential expression analysis of immune checkpoint genes between high-risk group and low-risk group. TMB = tumor mutation burden.

Immunotherapy has been a promising treatment in CRC, and evidences implied that TMB was closely related to sensibility of immunotherapy. In present study, TMB was significantly higher in the low-risk group (*P* = .018) (Fig. 7A). And risk score negatively correlated with degree of TMB, significantly (Cor = −0.16 and *P* < .001) (Fig. 7B). To further explore the immunotherapy potential of the different risk groups, differential expression analysis of immune checkpoint genes between risk groups was utilized. As demonstrated in Figure 7C, only HAVCR2 was significantly different between high-risk group and low-risk group (*P* < .05).

### 3.5. Correlation of the risk score and tumor immune microenvironment

**Figure 8.**
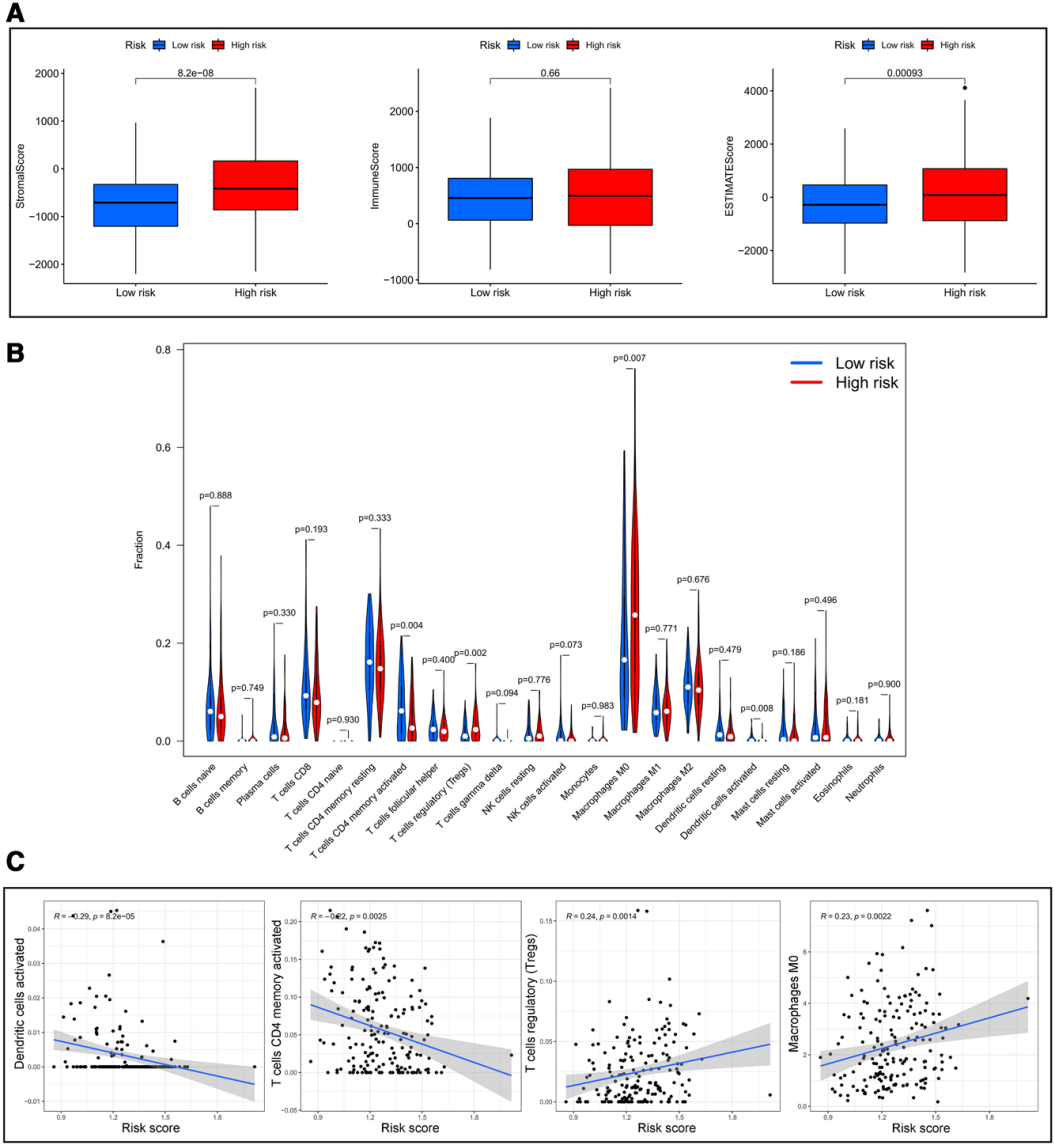
Risk score was associated with tumor immune microenvironment. (A) The boxplot showed the difference of risk score in stromal score, immune score, and ESTIMATE score, respectively. (B) Cellular fraction of different immune cells between high- and low-risk groups in TCGA datasets. (C) The correlation among risk score and activated dendritic cells, activated memory CD4^+^ T cells, regulatory T cells and M0 macrophages, respectively. TCGA = The Cancer Genome Atlas.

The ESTIMATE algorithm was applied to investigate the correlation of the risk score and the immune microenvironment in CRC. Next, Stromal score, Immune score, and ESTIMATE score of all CRC patients were calculated. As is Figure 8A showed, low-risk group had lower Stromal score and ESTIMATE score compared to the high-risk group (all *P* < .001). However, there was no significant difference in Immune scores (*P* = .66). The results indicated immune cells might enrich in high-risk group more than low-risk group.

To identify which immune cells take part in immune response of CRC, CIBERSORT algorithm was used to investigate the correction of risk score and 22 immune cell type (Fig. 8B and C). The results showed the significant differences in activated dendritic cells, activated memory CD4^+^ T cells, regulatory T cells and M0 macrophages. The proportions of activated dendritic cells (*P* = .008) and activated memory CD4^+^ T cells (*P* = .004) were higher in low-risk group. In contrast, regulatory T cells (*P* = .002) and M0 macrophages (*P* = .007) was higher in high-risk group. What’s more, risk score was positively correlated with regulatory T cells (Cor = 0.24 and *P* = .0014) and M0 macrophages (Cor = 0.23 and *P* = .0022). However, risk score was negatively correlated with activated dendritic cells (Cor = −0.29 and *P* < .001) and regulatory T cells (Cor = −0.22 and *P* = .0025). The above results revealed that the miRNAs constructing risk score might regulate the tumor immune microenvironment via suppressing or collecting these immune cell types, which led to different response to immunotherapy.

### 3.6. Drug sensitivity analysis of the risk score

**Figure 9.**
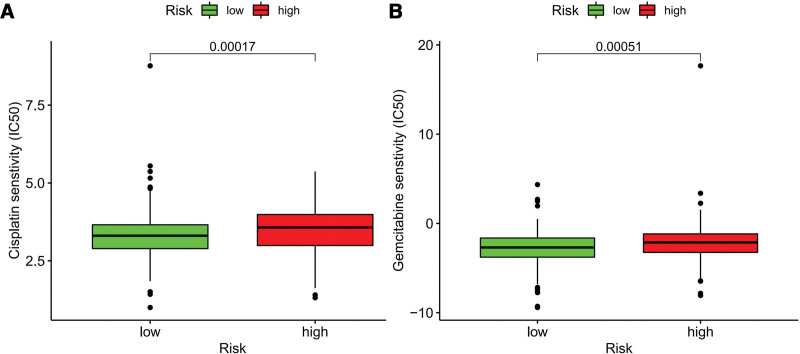
The IC50 of cisplatin (A) and gemcitabine (B) in high- and low-risk groups. IC50 = half-maximal inhibitory concentration.

The “pRRophetic” package was performed to investigate the correction between drug sensitivity and risk score. IC50 of cisplatin was lower in low-risk group compared to high-risk group (*P* < .001) (Fig. 9A). Besides, IC50 of gemcitabine was also lower in low-risk group compared to high-risk group (*P* < .001) (Fig. 9B).

### 3.7. The expression of the miRNAs

**Figure 10.**
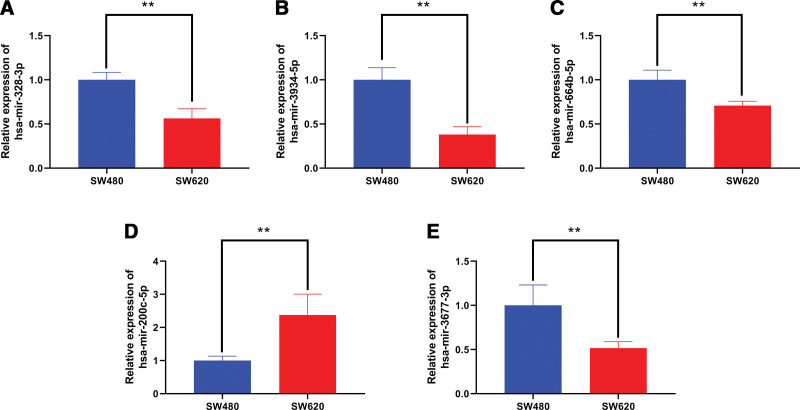
The expression of the 5 miRNAs in risk score was compared by qRT-PCR between SW480 and SW620. (A) miR-328-3p. (B) miR-3934-5p. (C) miR-664b-5p. (D) miR-200c-5p. (E) miR-3677-3p.

As the 5 miRNAs in risk score were related with the CRC prognosis, we suspected that the miRNAs were related to CRC metastasis. Therefore, the expression of the miRNAs was explored between SW480 and SW620, which were respectively separated from primary tumor and metastatic tumor of CRC. The results showed that miR-328-3p, miR-3934-5p, miR-664b-5p, miR-200c-5p and miR-3677-3p were down-regulated in SW620 compared with SW480, in contrast, miR-200c-5p was up-regulated in SW620 (Fig. 10).

## 4. Discussion

It’s known that CRC is a common malignant tumor with high incidence and mortality.^[1]^ There is still much challenge to investigate the mechanisms of CRC. Accumulating studies have showed that m6A modification played an important role in the occurrence, development and metastasis of CRC. METTL3 was found to be up-regulated in CRC and could maintained the expression level of SOX2 via m6A reader IGF2BP2 leading to tumor progression.^[20]^ Another study reported that FTO up-regulated the expression of ARL5B by inhibiting miR-181b-3p and promoted the invasion and migration of breast cancer.^[21]^ In this study, a total of 29 m6A-related miRNAs were found to be associated with survival. To explore the interaction and mechanisms of the miRNAs in CRC, KEGG enrichment analysis was performed and showed that the miRNAs were enriched in MAPK signaling pathway, Ras signaling pathway, Wnt signaling pathway, Hippo signaling pathway, TGF-β signaling pathway, etc. There was a study showed that downregulation of miR-874-3p could promote chemotherapeutic resistance in CRC via inhibiting Hippo signaling pathway.^[22]^ And miR-6887-3p could suppress MAPK signaling pathway via targeting Mex3a and promote oncogenesis of CRC.^[23]^ However, there is still rare study to explore the mechanisms between these pathways and the m6A-related miRNAs. This study may provide the direction to investigate the relationship of m6A modification and the miRNAs in CRC.

In previous studies, miRNAs have been proved to be good prognostic biomarkers in various cancers. The study reported that immune-related miRNA signature could be used to effectively predict the prognosis of head and neck squamous cell carcinomas.^[24]^ Another study identified a miRNA signature to predict the lymph node metastasis in CRC.^[25]^ However, the prognostic value of the m6A-related miRNAs in CRC remains to be clarified. Present study identified 5 m6A-related miRNAs (miR-328-3p, miR-3934-5p, miR-664b-5p, miR-200c-5p miR-3677-3p) and constructed a risk score via LASSO regression. The risk score exhibited potent value to predict the prognosis of CRC patients and was proved to be an independent adverse prognostic factor in CRC. Besides, the result suggested that the nomogram combined with risk score and clinicopathological parameters might be an effective tool in clinical application. Therefore, present study indicated m6a-related miRNAs could be used to effectively predict the prognosis of CRC patients.

It’s well known that CRC metastasis was a crucial prognostic factor, but the roles of the 5 miRNAs in CRC metastasis were not clear. Due to this, the expression levels of the 5 miRNAs were compared between SW480 and SW620. The result showed that the miRNAs were all down-regulated in SW620 expect for miR-200c-5p, which indicated these miRNAs might participate in CRC progression and metastasis. Studies have reported that miR-328-3p was down-regulated in CRC and could inhibit CRC proliferation and metastasis via inhibiting the PI3K/Akt signaling pathway.^[26]^ miR-3934-5p was also found to be a tumor suppressor in non-small cell lung cancer, and could enhance the sensitivity of tumor cells to cisplatin by targeting TP53INP1.^[27]^ Besides, miR-664b-5p and miR-200c-5p both could suppress the development of hepatocellular carcinoma through targeting AKT2 and MAD2L1, respectively.^[28,29]^ Additionally, the study showed that miR-3677-3p could promote hepatocellular carcinoma progression and metastasis via inhibiting GSK3β.^[30]^ There is still little study to explore the mechanisms between above 5 miRNAs and CRC metastasis. The miRNAs might be potential therapeutic targets for CRC and need to be investigated in the future.

Immunotherapy is a novel anti-cancer method and has been a focal point in various cancers, recently. However, CRC is regard as a cold tumor, which less responses to immunotherapy.^[31]^ Previous studies have proved that higher TMB indicated better effect in immunotherapy.^[32]^ In this study, risk score was negatively correlated with TMB, which indicated that lower risk score might be benefit from immunotherapy. Besides, the expression level of HAVCR2, an immune checkpoint gene, was found to be significantly different between risk groups. HAVCR2 is an activation induced inhibitory molecule involved in immune tolerance and has been reported to induce T cell exhaustion in CRC.^[33]^ Many researches reveled that targeting HAVCR2 is a promising treatment method and block of HAVCR2 could reduce tumor progression.^[34,35]^ These findings suggested that the risk score may be a promising predictor to predict the immunotherapy efficacy of CRC patients. And HAVCR2 might be a valuable therapeutic target in the future.

To date, immune microenvironment was considered to be an important role in development and treatment in cancers. Recently, accumulating studies has revealed that m6A methylation could regulate tumor immune microenvironment, which led to occurrence and progression in various cancers.^[36]^ In this study, we found that risk score was related with Stromal and Estimate score. The findings suggested the miRNAs in risk score may take part in regulation of tumor immune microenvironment in CRC. Activated dendritic cells, activated memory CD4^+^ T cells, regulatory T cells and M0 macrophages were also identified via CIBERSORT analysis. Infiltration of different immune cells could result in different outcome of tumor cells. Dendritic cells were reported to be associated with survival in colon cancer.^[37]^ Besides, regulatory T cells, memory CD4^+^ T cells and M0 macrophages could suppress the development of tumor.^[38]^ In the future, immune-activating strategies might be a novel treatment in CRC patients. And our findings could provide the theoretical support to investigate the roles of immune cells in CRC.

Since studies reported miRNAs could affect drug resistance,^[39]^ drug sensitivity analysis was also performed in this study. The outcome reveled that low-risk group may be more sensitive in cisplatin and gemcitabine. The results showed that risk score could classify patients under system treatment and provide the decision recommendation for clinicians.

However, there are several limitations should be considered in this study. Firstly, risk score still needs to verify in more external cohort. Secondly, the clinical information of TCGA cohort is not complete, so some CRC samples were excluded, which might affect research results.

## 5. Conclusion

Present study is the first to investigate the prognostic value of m6A-related miRNAs and constructed a risk score for further application. The risk score was correlated with TMB and immune cells infiltration, which could provide the important information in immunotherapy but also the direction to explore the mechanisms among immune cells and CRC. To sum up, the results suggested that m6A-related miRNAs could be a good prognostic predictor and provided novel insights into immunotherapy in CRC patients.

## Acknowledgments

The authors thank the public availability of the GEO (http://www.ncbi.nlm.nih.gov/geo/) and TCGA (https://tcga-data.nci.nih.gov/tcga) database so that the data can be freely used by us.

## Author contributions

**Conceptualization:** Xinze Qiu, Shiquan Liu.

**Data curation:** Da Chen, Shanpei Huang, Ni Chen.

**Formal analysis:** Da Chen, Shanpei Huang, Ni Chen.

**Investigation:** Da Chen, Jiangni Wu, Shengmei Liang.

**Visualization:** Peng Peng, Mengbin Qin.

**Writing – original draft:** Xinze Qiu, Shiquan Liu.

**Writing – review & editing:** Jiean Huang.

## Supplementary Material


