# Supplementary material for: Identification and verification of m6A-related miRNAs correlated with prognosis and immune microenvironment in colorectal cancer
Source: Medicine (Baltimore). 2023 Nov 17;102(46):e35984. doi: 10.1097/MD.0000000000035984 (PMC10659607; doi:10.1097/MD.0000000000035984)
Supplement: Supplementary file 1 [file medi-102-e35984-s001.docx]

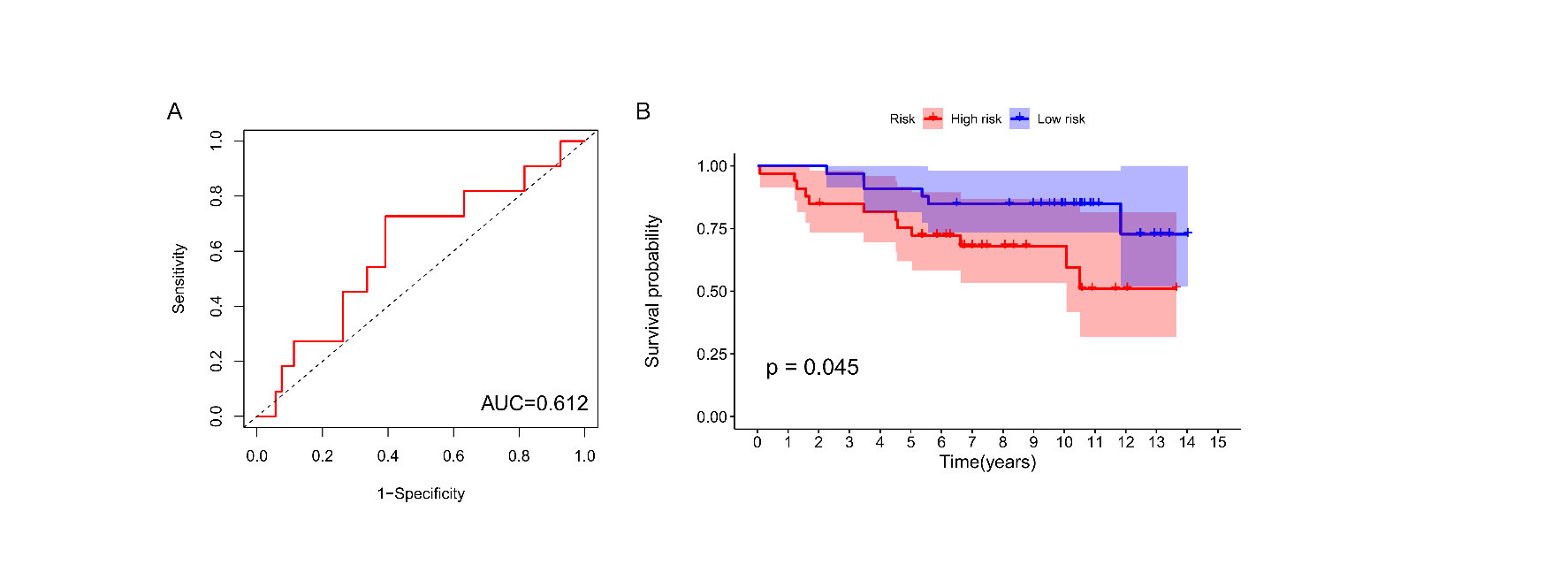


Supplemental **Fig. S1. (A)** ROC curves of risk score based on the five miRNAs to predict 5-year survival rate in GSE92928. **(B)** K-M survival curves of different risk groups were performed in GSE92928.
